# Exploring the associations between momentary cortisol levels and psychotic-like experiences in young adults: Results from a temporal network analysis of daily-life data

**DOI:** 10.1192/j.eurpsy.2024.1779

**Published:** 2024-09-20

**Authors:** Tomasz Grąźlewski, Jerzy Samochowiec, Hanna Gelner, Łukasz Gawęda, Bogna Bogudzińska, Krzysztof Kowalski, Patryk Piotrowski, Błażej Misiak

**Affiliations:** 1 Department of Psychiatry, Pomeranian Medical University, Szczecin, Poland; 2 Experimental Psychopathology Lab, Institute of Psychology, Polish Academy of Sciences, Warsaw, Poland; 3 Department of Psychiatry, Wroclaw Medical University, Wroclaw, Poland

**Keywords:** cortisol levels, ecological momentary assessment, hypothalamic–pituitary–adrenal axis, psychosis, psychotic-like experiences

## Abstract

Dysregulation of the hypothalamic–pituitary–adrenal (HPA) axis has been implicated in the development of psychosis and subthreshold psychotic symptoms commonly referred to as psychotic-like experiences (PLEs). The exact mechanisms linking the HPA axis responses with the emergence of PLEs remain unknown. The present study aimed to explore real-life associations between stress, negative affect, salivary cortisol levels (a proxy of the HPA axis activity) as well as PLEs together with their underlying cognitive biases (i.e., threat anticipation and aberrant salience). The study was based on the experience sampling method scheduled over 7 consecutive days in the sample of 77 drug-naïve, young adults (18–35 years). The saliva samples were collected with each prompt to measure cortisol levels. A temporal network analysis was used to explore the directed associations of tested variables. Altogether, 3234 data entries were analyzed. Data analysis revealed that salivary cortisol levels did not directly predict next-moment fluctuations of PLEs. However, higher salivary cortisol levels were associated with higher next-moment levels of PLEs through the effects on threat anticipation and negative affect. In turn, PLEs appeared to predict cortisol levels through the effects on negative affect and event-related stress. Negative affect and threat anticipation were the most central nodes in the network. There might be bidirectional associations between the HPA axis responses and PLEs. Threat anticipation and negative affect might be the most important mediators of these associations. Interventions targeting these mediators might hold promise for disrupting the connection between the HPA axis dysregulation and PLEs.

Psychotic-like experiences (PLEs) are subclinical psychotic symptoms that might precede the onset of psychosis and occur in the context of various mental disorders, not only those related to the psychosis spectrum. Their lifetime prevalence has been estimated at 5.8% [1]. It has been found that stress exposure might contribute to the emergence of PLEs through increasing negative affective responses, aberrant salience (defined as over-attribution of meanings to irrelevant stimuli), and threat anticipation (i.e., foreseeing future experiences as threatening) [2]

Stress exposure leads to the activation of the hypothalamic–pituitary–adrenal (HPA) axis. There is evidence that aberrant activity of the HPA axis might play a role in the development of psychosis [3]. Indeed, cortisol may increase dopaminergic activity in several regions of the brain, especially within the mesolimbic system [3]. Therefore, it might be hypothesized that it contributes to the occurrence of PLEs and aberrant salience. Also, it has been shown that anticipation of stressful events might be associated with cortisol responses [4].

To our knowledge, the interrelationships between HPA axis activity, aberrant salience, threat anticipation, and PLEs have not been investigated so far. In this study, we aimed to explore whether momentary cortisol responses contribute to the emergence of PLEs. We hypothesized that cortisol levels predict the emergence of PLEs through their effects on negative affect, threat anticipation, and aberrant salience.

Participants were young adults (aged 18–35 years) with PLEs who did not meet the criteria for underlying psychotic disorders. Participants with psychotic disorders were not included as the focus of this study was on subclinical symptoms, that is, PLEs. The sample reported in this study was a part of a larger project investigating epigenetic regulation of the HPA axis activity. All participants did not receive any psychiatric treatment during their lifetime. Recruitment procedures were scheduled within a multistep process (see Supplementary Appendix and Supplementary Table 1 for details).

The study was approved by the Ethics Committees at the Institute of Psychology (Polish Academy of Sciences in Warsaw, Poland, approval: 16/VII/2022), Wroclaw Medical University (Wroclaw, Poland, approval: 129/2022), and Pomeranian Medical University (Szczecin, Poland, approval: KB-006/25/2022). All individuals provided written informed consent for participation.

Data were collected using the experience sampling method (ESM) has greatly improved our understanding of the role of contextual factors in mental disorders. It is based on multiple observations of participants, often using mobile devices, that provide insights into daily experiences, minimizing the risk of a recall bias. Participants were assessed using the ESM questionnaires during seven consecutive days with a random sampling (six prompts per day emitted by the *Movisens app* between 9 a.m. and 10 p.m.). After each prompt, they were asked first to respond to the ESM questionnaire and then to collect saliva samples using a cotton swab (Salivette, Sarstedt, the Netherlands). Moreover, participants were asked to record the date and time of saliva collection. They stored saliva samples in their home freezer for the duration of the study. Next, saliva samples were transferred to research units and stored at –20°C. Responses and saliva samples provided more than 15 min. After a prompt was not considered in the data analysis. Participants received incentives equivalent to 250 EUR in case of a response rate of at least 80%.

The ESM questionnaires included 39–42 items. In this study, the analysis covered items measuring PLEs, negative affect, aberrant salience, threat anticipation, various categories of psychosocial stress, and salivary cortisol levels (Supplementary Table 2). The internal consistency of multiple-item constructs was acceptable-to-good.

To address our hypotheses, we approached a temporal network analysis [5]. This approach enables to explore time-series data without a predefined model of causality. Moreover, it allows for the investigation of multiple predictions in a single model, controlling for the effects of all variables in the network. It typically needs two assumptions to be met, that is, at least 20 observations per participant and data stationarity [5]. In our study, each participant completed 38–42 assessments. To test the second assumption, the Augmented Dickey–Fuller (ADF) test was used to analyze if the means and variance of the data depend on stationarity. Results of the ADF tests demonstrated that all variables met the assumption of stationarity (all *p*-values < 0.05). The temporal network analysis provides the opportunity to test predictions within specific time lags. It allows to test as to whether a variable at a certain timepoint *t* is predicted by the same variable (autoregression effects) and all other variables in the network at the preceding timepoint (*t* − 1). Finally, node centralities were assessed. Due to the fact that negative predictions were observed, the node expected influence was used. It shows the sum of edge weights directly connected to a specific node taking into consideration the presence of negative edges. Out-expected influence provides information on how strongly a specific node predicts other nodes, while in-strength presents on how strongly the node is predicted by other nodes directly connected to it. All analyses were conducted in the R software (packages: *mlVAR*, *qgraph*, and *psych*).

Altogether, 77 individuals (aged 24.6 ± 4.6 years, 80.5% women, Supplementary Table 3) participated in the present study (3234 data entries). Participants did not receive any psychiatric treatments during their lifespan and did not meet the criteria of psychotic disorders (Supplementary Table 3). All of them had a negative lifetime history of chronic physical health impairments that might affect the HPA axis (e.g., endocrine disorders, chronic inflammatory diseases, and autoimmune diseases). Descriptive characteristics of ESM constructs are provided in Supplementary Table 4. Almost all nodes (except for event-related stress) showed significant autocorrelations meaning that the variables at the timepoint *t* were predicted by themselves at timepoint *t* − 1 (Figure 1). Salivary cortisol levels did not directly predict next-moment fluctuations of PLEs. However, higher salivary cortisol levels were associated with higher next-moment levels of PLEs through the effects on threat anticipation and negative affect. In turn, PLEs appeared to predict cortisol levels through the effects on negative affect and event-related stress. Apart from lifestyle variables, salivary cortisol levels were directly predicted by previous-moment event-related stress (positive correlation) and area-related stress (negative correlation). It is also important to note that higher salivary cortisol levels directly predicted higher levels of aberrant salience and activity-related stress. In turn, aberrant salience was not found to predict next-moment levels of PLEs. However, PLEs were observed to predict next-moment aberrant salience through the effects on negative affect. Threat anticipation had the highest in-expected influence, while the highest out-expected influence was found for negative affect.

**Figure 1.**
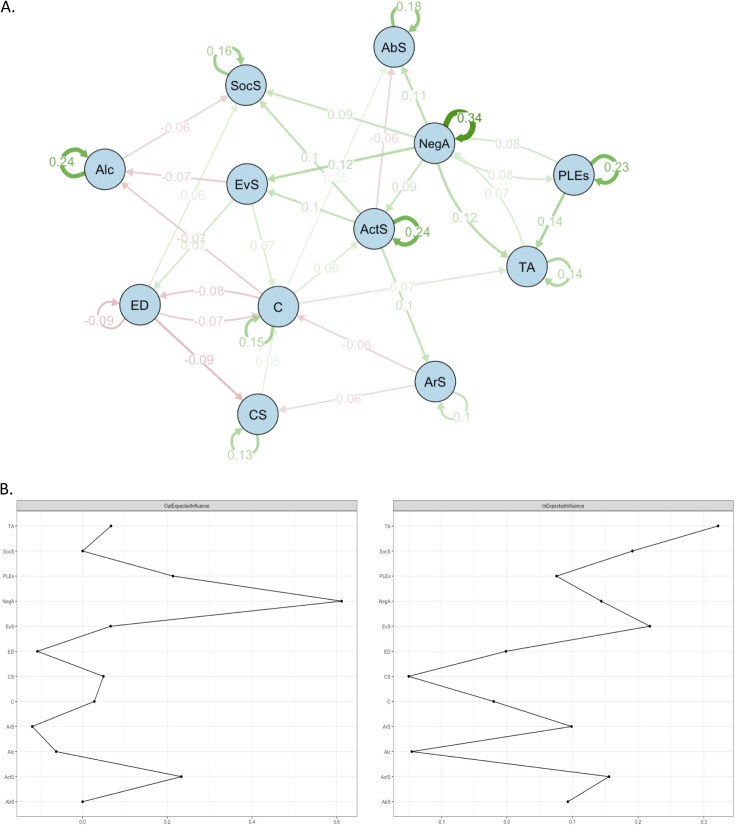
The temporal network analyzed in the present study (A) and corresponding nodes centrality metrics (B). Variables are visualized as nodes that are connected with edges showing directions of prediction. All visualized edges refer to significant predictions. Thicker and more saturated edges refer to stronger associations. AbS, aberrant salience; ActS, activity-related stress; Alc, alcohol use; ArS, area-related stress; C, salivary cortisol levels; CS, using caffeine and/or smoking; ED, eating and drinking; EvS, event-related stress; NegA, negative affect; PLEs, psychotic-like experiences; SocS, social stress; TA, threat anticipation.

The main findings indicate that elevated cortisol responses might contribute to the occurrence of PLEs through the effects on threat anticipation and negative affect. Both mediators, that is, threat anticipation and negative affect had the highest in- and out-expected influence centrality, respectively. However, a reversed causation also needs to be considered. Indeed, we also observed that PLEs may increase salivary cortisol levels through the effects on negative affect and appraisals of daily-life events as stressful. Notably, only two categories of daily-life stressors were found to predict salivary cortisol levels (event- and area-related stress).

Previous ESM studies have provided interesting insights into salivary cortisol responses across the psychosis spectrum. For instance, one study demonstrated that increases in salivary cortisol levels are significantly associated with increases in PLEs and negative affect among siblings of individuals with psychosis [6]. Moreover, this study demonstrated higher diurnal cortisol levels and elevated cortisol response to daily stressors in relatives compared to healthy controls. However, the diurnal cortisol slope did not differ significantly between both groups. The same group of researchers also revealed that siblings of individuals with psychosis showing small hippocampal volumes demonstrate increased emotional and cortisol stress reactivity compared to those with large hippocampal volumes [7]. Another study demonstrated no significant differences in overall diurnal cortisol levels between medicated and unmedicated individuals with psychosis, first-degree relatives of individuals with psychosis, and healthy controls [8]. Nevertheless, attenuated cortisol response to stress was observed in both groups of patients compared to healthy controls.

The relationship between cortisol responses and threat anticipation has been observed in non-clinical samples. It has been reported that physiological adjustments to anticipated stressors are associated with an increased release of cortisol [9]. However, the extent of cortisol release during threat anticipation depends on individual expectancies to deal with the future situation. Optimizing expectations (e.g., through positive feedback about individual abilities to cope or cognitive reappraisal) and distraction before stress may reduce cortisol responses [10]. Moreover, lower stress-induced cortisol responses have been observed in people with a better anticipatory stress regulation before experimental exposure [11]. These findings suggest that the release of cortisol related to threat anticipation might be influenced by interventions, for example, those that promote coping mechanisms and resilience. Finally, it is needed to note that both threat anticipation and negative affect were found to mediate the association between momentary stress and PLEs [2].

There are important strengths of this study, that is, the inclusion of drug-naïve individuals, high response rates in the ESM protocol, and multiple assessments of cortisol levels (up to 42 in each participant). However, some limitations also need to be pointed out and include low sample size, diagnostic heterogeneity, small partial correlation coefficients, the predominance of female participants, limited insights into causality due to observational type of findings, and a subjective character of ESM responses. Also, we did not thoroughly address the effects of lifestyle variables (e.g., sleep patterns, dietary habits, and exercise activity) that might be of importance due to a circadian rhythm of cortisol secretion. Finally, our sample was limited to participants aged 18–35 years. However, our decision to focus on this age group was due to the fact that PLEs are more likely to be observed in younger individuals [12]. These limitations are needed to be addressed by future studies. Nevertheless, the findings indicate that threat anticipation and negative affect are important mediators in the prediction of PLEs by cortisol responses. Moreover, our observations suggest that PLEs may also predict increases in cortisol levels through affective responses and negative appraisals of contextual factors. The observation that negative affect and threat anticipation are the most important variables provides premises for developing potential interventions for individuals with PLEs.

## Supporting information

Grąźlewski et al. supplementary material Grąźlewski et al. supplementary material

## Data Availability

Data generated in the present study (file name: “cortisol_ples.txt”) are available in the Open Science Framework (OSF) database (https://doi.org/10.17605/OSF.IO/8JU6B).

## Supplementary material

The supplementary material for this article can be found at http://doi.org/10.1192/j.eurpsy.2024.1779.
