# Supplementary material for: Exploring the associations between momentary cortisol levels and psychotic-like experiences in young adults: Results from a temporal network analysis of daily-life data
Source: Eur Psychiatry. 2024 Sep 20;67(1):e54. doi: 10.1192/j.eurpsy.2024.1779 (PMC11457160; doi:10.1192/j.eurpsy.2024.1779)
Supplement: Grąźlewski et al. supplementary material [file S0924933824017796sup001.docx]

**Supplementary Appendix**

***Recruitment procedures***

First, an internet-based survey was implemented between April and October 2022. Altogether, 4203 individuals from three large Polish cities participated in the survey. Findings from the survey were published elsewhere [1-4]. The survey included 16 items to measure the presence of PLEs in the preceding month (Supplementary Table 1). Three questionnaires were used to develop the survey, including the Revised Hallucination Scale (RHS) [5-7], the Revised Green et al. Paranoid Thoughts Scale (GPTS) [8], and the Prodromal Questionnaire–16 (PQ–16) [9]. Also, the screening questions from the Mini-International Neuropsychiatric Interview (M.I.N.I.) [10]were used.

Subsequently, telephone interviews with subjects showing the highest scores in the questionnaire recording PLEs were conducted. To provide further validation of PLEs, selected questions from the Comprehensive Assessment of At Risk Mental States (CAARMS) [11, 12] were used and covered the following symptoms: 1) ideas of reference (“Have you felt that things that were happening around you had a special meaning, or that people were trying to give you messages?”, “What is it like?”, and “How did it start?”); 2) suspiciousness and persecutory ideas (“Has anybody been giving you a hard time or trying to hurt you?”, “Do you feel like people have been talking about you, laughing at you, or watching you?, “What is it like?”, and “How do you know this?”) and 3) hallucinations (“Do you ever hear things that may not really be there?, “Do you ever hear things that other people seem not to, such as sounds, or voices?”, “What do you hear?”, “At the time you hear these things, how real do they seem?”, “Do you realize they are not real at the time, or only later?”). Individuals with a positive screening for the current episode of major depression and substance use disorders were interviewed for their presence using the M.I.N.I.. Participants were included in the next recruitment stage in case of meeting the following criteria: 1) age: 18 – 35 years; 2) a negative lifetime history of psychiatric treatment and 3) the global rating for ideas of reference and/or suspiciousness and persecutory ideas of 3 – 5 points, and/or the global rating for hallucinations of 3 – 4 points (i.e., below the psychosis threshold). In turn, the following exclusion criteria were used: 1) the current severe episode of major depression and 2) a diagnosis of substance use disorder (except for nicotine dependence).

Finally, interviews with full versions of the M.I.N.I. and CAARMS were performed by trained psychologists and psychiatrists in-person.

***Assessment of salivary cortisol levels***

Cortisol levels were determined based on the electrochemiluminescence method (the Cobas Pro e801, Roche). The CORTISOL II Elecsys Reagent for Roche (reference number: 06687733190) was used. The coefficients of variation for repeatability, intermediate precision, and reproducibility for saliva were ≤ 4.4 %, ≤ 10.9 %, and ≤ 11.4 %, respectively [40]. Cortisol levels were assessed by the commercial laboratory (ALAB, Warsaw, Poland).

**Supplementary Table 1.** The items used to record psychotic-like experiences at the screening stage. All items were scored on a 4-point scale (1 – “never”; 2 – “sometimes”; 3 – “often” and 4 – “almost always”). Participants were asked to score those experiences that had not been related to substance use and were present over the preceding month. The total score was between 16 and 64. The Cronbach’s alpha was 0.811 in our sample (*n* = 4203).

| Item | Content | Questionnaire |
| --- | --- | --- |
| 1 | “I hear voice speaking my thoughts aloud” | RHS |
| 2 | “I hear people call my name and find that nobody has done so” |  |
| 3 | “I see shadows and shapes when there is nothing there” |  |
| 4 | “I spent time thinking about friends gossiping about me” | R-GPTS |
| 5 | “People wanted me to feel threatened, so they stared at me” |  |
| 6 | “I was convinced there was a conspiracy about me” |  |
| 7 | “I was distressed by being persecuted” |  |
| 8 | “People have been dropping hints for me” |  |
| 9 | “When I look at a person, or look at myself in a mirror, I have seen the face change right before my eyes” | PQ-16 |
| 10 | “I have heard things other people can’t hear like voices of people whispering or talking” |  |
| 11 | “I often feel that other have it in for me” |  |
| 12 | “I have seen things that other people apparently can’t see” |  |
| 13 | “I have had the sense that some person or force is around me, even though I could not see anyone” |  |
| 14 | “I sometimes see special meanings in advertisements, shop windows, or in the way things are arranged around me” |  |
| 15 | “I sometimes smell or taste things that other people can’t smell or taste” |  |
| 16 | “I often seem to live through events exactly as they happened before” |  |

*Abbreviations:* PQ-16, the Prodromal Questionnaire-16 [9]; R-GPTS, the Revised Green Paranoid Thoughts Scale [8]; RHS, the Revised Hallucination Scale [5-7]

**Supplementary Table 2.** The ESM items used in the present study.

| Domain | Items | Cronbach’s alpha | |
| --- | --- | --- | --- |
|  |  | Within-subjects | Between-subjects |
| Aberrant salience | “Everything grabs my attention right now”  “Everything seems to have meaning right now”  “I notice things that I haven’t noticed before” | 0.72 | 0.92 |
| Threat anticipation | “I think that something unpleasant will happen” | – | – |
| Event stress | “Think about the most important event since the last beep (“during the last two hours” in case of the first beep during specific day). This event was…”. | – | – |
| Area-related stress | “I feel being in this neighborhood unpleasant” | – | – |
| Activity-related stress | “I would prefer doing something else”  “My current activity is difficult to me”  “My current activity is pleasant to me” (reversed scoring) | 0.82 | 0.84 |
| Social stress | “Whom are you staying with right now?”  “I would prefer to be alone (if with someone)/I would prefer to have company (if alone)”  “I find being with these people pleasant (if with someone)/it is pleasant to be alone (if alone)” (reversed scoring) | 0.81 | 0.75 |
| Negative affect | “I feel anxious”  “I feel down”  “I feel insecure”  “I feel annoyed”  “I feel lonely” | 0.80 | 0.86 |
| PLEs | “My thoughts are so strong that I can almost hear them”  “I hear things that aren’t really there”  “I see things that aren’t really there”  “I have the sense that some person or force is around me although I can’t see anyone”  “I see special meanings in advertisements, shop windows, or in the way things are arranged around me”  “I am confused whether something I experienced was real or imaginary”,  “My thoughts are influenced by others”  “I can’t get these thoughts out of my head” | 0.72 | 0.88 |
| Lifestyle behaviors | “Since the last beep, have you smoked cigarettes or drink coffee?”)  “Since the last beep, have you eaten or drunk non-alcoholic beverages?”)  “Since the last beep, have you drink beverages containing alcohol?” | – | – |

The majority of items were rated on a 7-point scale (from 1 – “not at all” to 7 – “very much”), except for the first item to assess social stress (“whom are you staying with right now?”; scoring: 1 – partner, 2 – family, 3 – friends, 4 – colleagues, 5 – acquaintances, 6 – strangers, 7 – others, and 8 – nobody). Also, in case of the following item: “think about the most important event since the last beep (“during the last two hours” in case of the first beep during specific day). This event was…” the scoring was between 1 – very unpleasant to 7 – very pleasant (reversed scoring was used). Almost all items in the ESM questionnaire were used by the prior studies [37 - 39]. Three additional items were derived from the PQ-16 [33]: “I have the sense that some person or force is around me although I can’t see anyone”. The items used to record lifestyle behaviors were not used by previous studies (yes-or-no responses). In case of multiple-item constructs, mean scores were calculated.

**Supplementary Table 3.** General characteristics of the sample (*n* = 77).

|  | Mean ± SD or *n* (%) |
| --- | --- |
| Age, years | 24.6 ± 4.6 |
| Sex, males | 15 (19.5) |
| Education, years | 15.1 ± 2.6 |
| Education level  Higher  Student  Secondary  Vocational  Primary | 31 (40.2)  16 (20.8)  26 (33.8)  1 (1.3)  3 (3.9) |
| Occupation  Employed (full-time) or student  Employed (part-time)  Unemployed | 56 (72.7)  14 (18.2)  7 (9.1) |
| PLEs, screening score | 33.9 ± 6.2 |
| CAARMS, subclinical positive symptoms^a^ | 14.5 ± 6.7 |
| Psychiatric diagnosis  Any anxiety disorder, current  Depression, current  Bipolar disorder  OCD, current  PTSD  Eating disorder, current | 38 (49.3)  13 (16.9)  7 (9.1)  1 (1.3)  8 (10.4)  10 (13.0) |
| ESM – compliance (questionnaires)^b^, % | 99.0 ± 2.0 |
| ESM – valid saliva samples^c^, % | 82.0 ± 13.0 |
| ESM – delay in responding (questionnaires), min. | 1.3 ± 3.2 |
| ESM – delay in providing valid saliva samples, min. | 3.9 ± 9.0 |
| ESM – time lag between beeps, min. | 137.6 ± 48.5 |

^a^the sum of scores obtained for the severity and frequency of positive symptoms across all subscales, i.e., unusual thought content, non-bizarre ideas, perceptual abnormalities, and disorganized speech (range: 0 – 48)

^b^the ratio of the number of self-evaluations completed by the participants over the maximum number of self-evaluations allowed by the protocol

^c^the ratio of the number of valid saliva samples provided by the participants over the maximum number of saliva samples allowed by the protocol (valid saliva samples were those provided within 15 min. after each beep with detectable cortisol levels)

*Note:* CAARMS, the Comprehensive Assessment of At Risk Mental States; ESM, the experience sampling method; OCD, obsessive-compulsive disorder; PLEs, psychotic-like experiences; PTSD, post-traumatic stress disorder

**Supplementary Table 4.** Intra-individual characteristics of constructs measured using the ESM. Scores across all variables assessed using the ESM questionnaires ranged between 1 and 7.

|  | M | SD |
| --- | --- | --- |
| PLEs | 1.99 | 1.02 |
| Negative affect | 3.03 | 1.51 |
| Event-related stress | 3.58 | 1.63 |
| Activity-related stress | 3.58 | 1.75 |
| Area-related stress | 2.67 | 1.84 |
| Social stress | 3.27 | 1.85 |
| Aberrant salience | 2.63 | 1.57 |
| Threat anticipation | 2.83 | 1.96 |
| Cortisol levels | 1.28 | 1.47 |

*Note:* M, mean; PLEs, psychotic-like experiences; SD, standard deviation

**References**

[1] Misiak B, Szewczuk-Boguslawska M, Samochowiec J, Moustafa AA, Gawęda Ł. Unraveling the complexity of associations between a history of childhood trauma, psychotic-like experiences, depression and non-suicidal self-injury: A network analysis. J Affect Disord. 2023. <https://doi.org/https://doi.org/10.1016/j.jad.2023.05.044>.

[2] Misiak B, Gaweda L, Moustafa AA, Samochowiec J. Insomnia moderates the association between psychotic-like experiences and suicidal ideation in a non-clinical population: a network analysis. Eur Arch Psychiatry Clin Neurosci. 2024;274(2):255-63. <https://doi.org/10.1007/s00406-023-01653-3>.

[3] Misiak B, Samochowiec J, Gaweda L, Frydecka D. Association of sociodemographic, proximal, and distal clinical factors with current suicidal ideation: Findings from a nonclinical sample of young adults. Eur Psychiatry. 2023;66(1):e29. <https://doi.org/10.1192/j.eurpsy.2023.14>.

[4] Misiak B, Frydecka D, Kowalski K, Samochowiec J, Jablonski M, Gaweda L. Associations of neurodevelopmental risk factors with psychosis proneness: Findings from a non-clinical sample of young adults. Compr Psychiatry. 2023;123:152385. <https://doi.org/10.1016/j.comppsych.2023.152385>.

[5] Gaweda L, Kokoszka A. [Polish version of the Revised Hallucination Scale (RHS) by Morrison et al. Its factor analysis and the prevalence of hallucinatory-like experiences among healthy participants]. Psychiatr Pol. 2011;45(4):527-43.

[6] Morrison AP, Wells A, Nothard S. Cognitive and emotional predictors of predisposition to hallucinations in non-patients. Br J Clin Psychol. 2002;41(Pt 3):259-70. <https://doi.org/10.1348/014466502760379127>.

[7] Morrison AP, Wells A, Nothard S. Cognitive factors in predisposition to auditory and visual hallucinations. Br J Clin Psychol. 2000;39(1):67-78. <https://doi.org/10.1348/014466500163112>.

[8] Freeman D, Loe BS, Kingdon D, Startup H, Molodynski A, Rosebrock L, et al. The revised Green et al., Paranoid Thoughts Scale (R-GPTS): psychometric properties, severity ranges, and clinical cut-offs. Psychol Med. 2021;51(2):244-53. <https://doi.org/10.1017/S0033291719003155>.

[9] Ising HK, Veling W, Loewy RL, Rietveld MW, Rietdijk J, Dragt S, et al. The validity of the 16-item version of the Prodromal Questionnaire (PQ-16) to screen for ultra high risk of developing psychosis in the general help-seeking population. Schizophr Bull. 2012;38(6):1288-96. <https://doi.org/10.1093/schbul/sbs068>.

[10] Sheehan DV, Lecrubier Y, Sheehan KH, Amorim P, Janavs J, Weiller E, et al. The Mini-International Neuropsychiatric Interview (M.I.N.I.): the development and validation of a structured diagnostic psychiatric interview for DSM-IV and ICD-10. J Clin Psychiatry. 1998;59 Suppl 20:22-33;quiz 4-57.

[11] Yung AR, Yuen HP, McGorry PD, Phillips LJ, Kelly D, Dell'Olio M, et al. Mapping the onset of psychosis: the Comprehensive Assessment of At-Risk Mental States. Aust N Z J Psychiatry. 2005;39(11-12):964-71. <https://doi.org/10.1080/j.1440-1614.2005.01714.x>.

[12] Jaracz J, Grzechowiak M, Raczkowiak L, Rataj K, Rybakowski J. [Polish version of Comprehensive Assessment of At Risk Mental States (CAARMS)--the description of the method]. Psychiatr Pol. 2012;46(1):95-107.
